# Reputation Revision Method for Selecting Cloud Services Based on Prior Knowledge and a Market Mechanism

**DOI:** 10.1155/2014/617087

**Published:** 2014-02-17

**Authors:** Qingtao Wu, Xulong Zhang, Mingchuan Zhang, Ying Lou, Ruijuan Zheng, Wangyang Wei

**Affiliations:** Information Engineering College, Henan University of Science and Technology, Luoyang 471023, China

## Abstract

The trust levels of cloud services should be evaluated to ensure their reliability. The effectiveness of these evaluations has major effects on user satisfaction, which is increasingly important. However, it is difficult to provide objective evaluations in open and dynamic environments because of the possibilities of malicious evaluations, individual preferences, and intentional praise. In this study, we propose a novel unfair rating filtering method for a reputation revision system. This method uses prior knowledge as the basis of similarity when calculating the average rating, which facilitates the recognition and filtering of unfair ratings. In addition, the overall performance is increased by a market mechanism that allows users and service providers to adjust their choice of services and service configuration in a timely manner. The experimental results showed that this method filtered unfair ratings in an effective manner, which greatly improved the precision of the reputation revision system.

## 1. Introduction

The rapid developments of cloud computing means that cloud services have become the main computing mode on the Internet. Many services have been deployed to provide similar functionalities. However, the problem of identifying reliable services has attracted the attention of researchers [1, 2]. Thus, the concepts of trust and reputation [3] have been introduced to assess the reliability of cloud services.

Reputation is a subjective assessment of a cloud service, which is based on individual experience or the recommendations of other users. Reputation and trust are dynamic, which makes the construction of an evaluation standard a challenging task. In addition, the occurrence of malicious evaluations [4, 5], deliberate praise, and the personal preferences of users means that a standardized reputation value may differ from the true value.

Recently, various reputation revision systems have been proposed to address the challenges posed by open and dynamic cloud service environments [6]. Most of these systems are focused on the calculation of reputation ratings, reputation management, experience, and other features of dynamic environments [7–10] that might provide an appropriate reference for users. However, the existence of unfair ratings greatly affects the accuracy of trust evaluations. Currently, these reputation models are mainly tending to the accuracy of trust evaluations [11, 12]; however, these existing methods are limited by personality preference.

Based on historical user evaluations and preferences related to specific requirements, we propose a method that revises the reputation rating. This prior knowledge is combined with a filtering algorithm based on the similarities of evaluations, which can distinguish between unfair ratings in an effective manner. This algorithm also includes a market mechanism that allows users and services to act as buyers and sellers. Our method uses only the user feedback ratings data related to a service. Extensive experiments showed that our method distinguished and filtered unfair ratings correctly, while it could also recommend appropriate services for a specific user based on their preferences in a dynamic market environment.

The remainder of this paper is organized as follows. In Section 2, we provide an overview of related work on reputation revision methods.  Section 3 describes the problem and provides definitions related to a specific scenario.  Section 4 explains our proposed reputation revision framework and Section 5 presents the experimental results. In Section 6, we conclude with the discussion and we summarize the contributions of this study.

## 2. Related Work

Reputation is a term that has different meanings in various domains. In our study, reputation is defined as an indicator of whether a user is willing to select a service based on the evaluations of other users. Thus, the result of a reputation evaluation will affect the decision about whether to interact with a service provider. Feedback related to previous interactions among users and service providers is collected by a reputation system to predict its future reliability [13, 14]. Reputation evaluations are essential parts of many recommendation systems [15]. Many systems are located in a central server, which can access, collect, and evaluate historical reputation scores from a large number of users [16–19]. Yang et al. [9] proposed a reputation management framework for service selection based on similarity theory. Different weights were set to compute the reputation based on the unique recommendations of users. However, the management framework was designed for a centrally controlled server, which was not suitable for a dynamic cloud service environment.

Several reputation evaluation approaches have been proposed for distributed systems. Ghaffarinejad and Akbari [10] introduced a distributed reputation mechanism, which was based on a number of special reputation centers. Each special reputation center collected reputation information for predetermined services offered by different service providers. However, this method was still somewhat centralized. Faniyi and Bahsoon [7] proposed a decentralized resource control mechanism, which introduced a market-oriented cloud computing architecture. However, the reputation system was vulnerable to whitewashing, incorrectly reported feedback, and collusion attacks (where several users coordinate their feedback to manipulate reputation information).

Kussul et al. [20] focused on the analysis of security threats in trust models and assessed the most important and critical security threats for a utility-based reputation model based on grids. Dong-Sheng et al. [21] proposed a distributed trust mechanism, which calculated two reputation values (for a seller and a buyer) for each node in an iterative manner based on the transaction history. This mechanism could rapidly reduce the reputation values of malicious nodes and prevent collusion attacks. To construct a trustworthy computing environment, Gui et al. [19] proposed a penalty-incentive mechanism based on a repeated game theory, which included a rule related to rewards and punishments. Hawa et al. [16] introduced enhanced reputation-based cooperation incentives, which facilitated better detection and control of free riders. This approach enhanced the scalability and fairness of the system.

Kim and Phalak [18] proposed a computational trust framework for predicting the degree of trust. In addition, Su et al. [12] developed a priority-based trust model, which determined the trustworthiness of a service provider based on designated referees and its historical performance. This method only used third-party evaluations of the previous overall performance of the service and did not consider the individual preferences of the current user. Gorner et al. [22] proposed several improvements for trust modeling, including limiting the size of the advisor network either by specifying the maximum size of a buyer's advisor network or by setting a minimum trustworthiness threshold for agents accepted into the advisor network.

Another previous study [23] proposed a method for revising reputation values that calculated the reputation based on the difference between the advertised quality of service (QoS) provided by service providers and the evaluations made by consumers. Next, the consumers were sorted based on the reputation ratings they provided and those consumers that might be involved with collusion were mined using an association rules algorithm. Finally, the updated reputation was recalculated and saved in the reputation center.

## 3. Overview

### 3.1. Problem Description and Scenario Definition

Our method was developed to overcome some of the limitations of existing reputation mechanisms and is suitable for open and dynamic environments. Before providing the details of our proposed reputation revision mechanism, it is necessary to define the scope of our study and to explain some of the definitions used in this paper.

**Figure 1:**
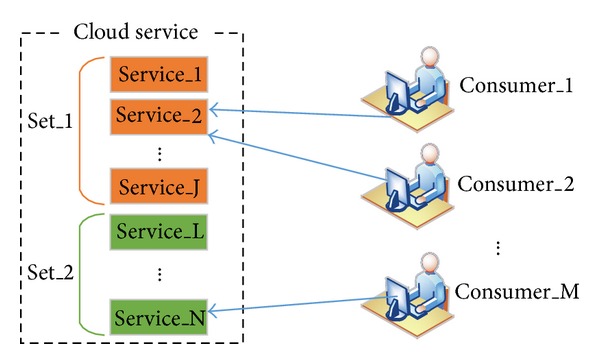
Cloud service selection model.

A cloud computing system provides services according to a third-party mechanism. Thus, users only need to be concerned with the service provided by the cloud. The cloud service selection model shown in Figure 1 contains two agent types, that is, consumer agents and service agents. Services that share the same functionality are placed in the same sets.

Users often select services from the same functional groups based on their own experiences and those of other consumers. However, unreliable evaluations may mean that the reputation of a service does not represent their actual reliability. Some service providers pay consumers to give them high scores for their services or to give low scores to their competitors. Furthermore, it may be difficult to satisfy users with specific requirements. These issues may mean that the service scores are not effective reference sources for users.

**Definition 1**

A service can be described as a 2-tuple; that is, Service = (Function, QoS). Function is a set of common properties, where different services are classified into separate sets. The main components of QoS include the response time (RT), cost (C), and reliability (R). In the present study, we define QoS as a 3-tuple; that is, QoS = (RT, C, R).

**Definition 2**

The rating score (RS) is derived from the distributed consumer agents. RT represents the degree of satisfaction with the service. RT is defined as a 3-tuple; that is, RS = (V_RT, V_C, V_R), where V_RT, V_C, and V_R are the values of the properties of the service given in Definition 1. We use percentile scores to distinguish between good and bad performance.

**Definition 3**

The consumer rating is the major focus, and the consumer is associated with multiple services from different providers. Thus, the consumer agent can be described as follows.

Consumer = (C_ID, ⋃_*i*_Service_*i*, ⋃_*j*_RS_*j*), where C_ID is the consumer's identifier and ⋃_*i*_Service_*i* and ⋃_*j*_RS_*j* are the services and rating scores associated with the consumer, respectively.

### 3.2. Overview of the Entire Reputation System

**Figure 2:**
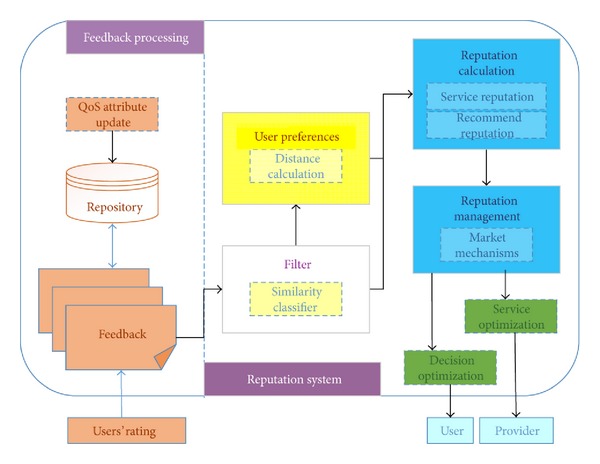
Reputation system framework.

In this section, we briefly introduce our reputation system structure. The framework of the reputation system is shown in Figure 2.

In general, the framework is applicable to most of the ratings-based experiences shared on online platforms where users evaluate services with numerical ratings. Figure 2 shows that the users ratings are collected as feedback to reputation processing node and the feedback is quantified based on the QoS attributes in the knowledge repository. After normalizing the feedback data, the ratings are filtered based on similarity classification. Next, we set the preferences for abnormal users, who may have specific service requirements. The user preferences mean that the recommendation system provides the most relevant services. There is also a certain degree of punishment for collusive users, who make the results confusing when service recommendations are required. The core of the system is the reputation calculation, where we combine the result from filtering with the historical reputation to generate a reputation value that is credible and reliable. The final part of the reputation framework is reputation management. Using a market mechanism, the service provider can optimize his service configuration and the user can optimize his decision. Thus, the service quality is optimized for the overall environment using the reputation system. Filtering abnormal users also makes the reputation of the service more accurate.

## 4. Reputation Revision Mechanism

In this section, we describe the mechanism used to ensure a more accurate level of consumer trust in dynamic environments. As mentioned in Section 3, consumers evaluate services using numerical ratings. The proposed method estimates the degree of trust a consumer places in a service based on the consumer's preference and it filters the abnormal reputation ratings.

### 4.1. Filtering the Abnormal Reputation Ratings

The presence of inaccurate assessments affects the overall evaluation of a service to some extent. Thus, we use the similarity to distinguish between abnormal evaluations, which reduces the effects of abnormal reputation ratings. The Euclidean distance is the shortest length of a line in n-dimensional space, which is usually defined as the real distance between two points in *n*-dimensional space.

We use *S* = {*s*_1_, *s*_2_,…, *s*_*n*_} to represent a service set where services share the same function. *C* = {*c*_1_, *c*_2_,…, *c*_*m*_} is the set of consumers. *P* = {*p*_1_, *p*_2_,…, *p*_*s*_} is the set of QoS parameters [24] for a service. We specify *q*_*ij*_ as the parameter values of *p*_*j*_ for *s*_*i*_, as shown in Table 1. *r*_*ij*_ represents the reputation of *s*_*j*_ in *c*_*i*_, where 0 ≤ *r*_*ij*_ ≤ 1, as shown in Table 2.

Each row is regarded as a node. Thus, the similarity between two nodes can be represented by the Euclidean distance. If the distance between two nodes is high, the similarity will obviously be low. Thus, we use the following to compute the similarity between the service parameter and a user's evaluation:

(1) $$\begin{array}{c} \text{similarity}=\frac{1}{\left(\sum_{1}^{n}{\left(X_{i}-Y_{i}\right)}^{2}/n\right)}. \end{array}$$

In (1), *X* and *Y* are vectors, where *X*_*i*_ (or *Y*_*i*_) represents the value of the *i*-dimension in the vector. To filter abnormal evaluations, we need to determine whether the parameters of the service configuration have changed. If the parameters of a service have changed, a distance will be generated. In general, there is no change in the configuration of the service parameters. However, if the parameters of the service configuration change, the historical reputation weighting will decline rapidly. If a consumer's evaluation is a considerable distance from the mean of all the other service evaluations made by consumers, we conclude that this consumer's evaluation is problematic and it must be filtered before further processing.

### 4.2. Estimation of Consumer Preference

All consumer evaluations are biased to some extent, so we need to set all of the abnormal consumer preferences. In this case, bias refers to subjective evaluations of objective factors by consumers that deviate from the norm. Consumers who make frequent unfair evaluations of services have extreme preferences. Malicious evaluation refers to the intentional denial of the objective facts, undeserved praise, or unmerited negative feedback. Thus, setting preferences allows us to eliminate abnormal evaluations. However, consumers can change their preferences if they have unusual requirements.

The preference weight (PW) represents a consumer's personal service preferences. A higher PW indicates that the rating for a service differs greatly from that of most users. The PW is constructed using two factors, that is, *C* Value and Ref. *C* Value represents a consumer's rating score, while Ref describes the contribution to the PW made by the total consumer scores. PW is defined using the following formula:

(2) $$\begin{array}{c} \text{PW}=C\;\text{Value}-\text{Ref}. \end{array}$$

*C* Value is the service's current reputation score given by a consumer. According to (3), Ref is the reference value for the overall score. The consumer rating scores have a normal distribution. Ref is the normal position parameter that describes the location of the central tendency of the normal distribution. rateScore = Ref is the normal to the axis of symmetry, which is completely symmetrical. In a normal distribution, the mean, median, and mode are the same; that is, they are equal to Ref. *σ* indicates the data distribution with a normal degree of dispersion:

(3) $$\begin{array}{c} f\left(\text{rateScore}\right)=\frac{1}{\sqrt{2\pi }\sigma }e^{{\left(\text{rateScore}-\text{Ref}\;\right)}^{2}/2\sigma^{2}}, \end{array}$$

(4) $$\begin{array}{c} \text{Ref}=\frac{1}{N}\overset{N}{\underset{i=1}{\sum }}\text{rateScor}{\text{e}}_{i}, \end{array}$$

(5) $$\begin{array}{c} \sigma =\sqrt{\frac{1}{N}\overset{N}{\underset{i=1}{\sum }}{\left(\text{rateScor}{\text{e}}_{i}-\text{Ref}\;\right)}^{2}.} \end{array}$$

**Figure 3:**
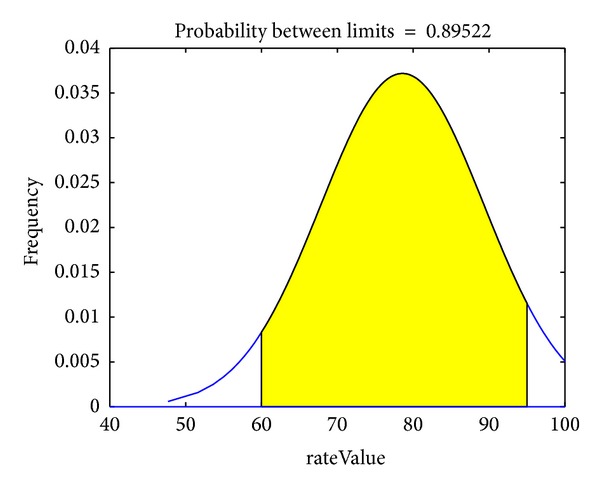
Distribution of a service's rateScore given by consumers.

The rateScore distribution of a service is shown in Figure 3 (the data used to produce the figure were based on the evaluation of a service). The PWs are set for overall consumer but they just apply to a small minority of consumers who have obviously deviation to the true quality of service level. According to Figure 3, we simply need to set separate PWs for the reputations that are outside the confidence interval. The confidence interval here refers to the proportion of real evaluation,which can be used as the reliability estimation that is usually defined by a large amount of observation. Obviously, an excessive proportion may mislead the service evaluation while a too little one leads to an unauthentic conclusion.

If *σ* and Ref are known, the service rateScore follows a normal distribution *N* (Ref, *σ*). We define the main interval using (6). *α* is the width of the main interval and *δ* is the probability of the evaluation given by most users:

(6) $$\begin{array}{c} P\left\{\left|\text{rateScore}-\text{Ref}\;\right|\leq f\left(\frac{\alpha }{2}\right)\right\}=\delta . \end{array}$$

Thus, we only need to set an appropriate value for *δ* to distinguish between abnormal evaluations. So we can give the abnormal ratings malicious preference treatment and reduce the weight of them in the whole calculation of service reputation. Suitable recommendations of appropriate services can be made based on the global reputation of a service and the preferences of users, while the system also punishes malicious users.

### 4.3. Calculation of the Integrated Reputation

The reputation rating of a cloud service given by a specific user is the weighted average of the directly experienced reputation and the historical reputation, which is obtained using (7). RoS_*ij*_ is the reputation rating based on the direct experience of service *j* given by consumer *i*. *C*_*ij*_ is the current reputation rating of service *j* given by consumer *i*. HsR_*ijk*_ is the *k*th reputation rating of the historic reputation rating given by consumer *i* to service *j*, and *l* is the total number of times that consumer *i* rates service *j*. *β* is the weight factor of the current reputation, 0 ≤ *β* ≤ 1

(7) $$\begin{array}{c} \text{Ro}{\text{S}}_{ij}=\left(1-\beta \right)C_{ij}+\frac{\beta }{l}\overset{l}{\underset{k=1}{\sum }}\text{Hs}{\text{R}}_{ijk}. \end{array}$$

We need to integrate the evaluations of the same service to calculate a service's reputation using the method proposed earlier. After calculating the mean score and the variance (using (8) and (9), resp.) of a service, we can determine its normal distribution (using (10)):

(8) $$\begin{array}{c} {\text{Ref}}_{j}=\frac{1}{r}\overset{r}{\underset{i=1}{\sum }}\text{Ro}{\text{S}}_{ij}, \end{array}$$

(9) $$\begin{array}{c} \sigma^{2}=\frac{1}{r}\overset{r}{\underset{i=1}{\sum }}{\left(\text{R}{\text{oS}}_{ij}-{\text{Ref}}_{j}\right)}^{2}, \end{array}$$

(10) $$\begin{array}{c} \text{Ro}{\text{S}}_{j}\sim \left({\text{Ref}}_{j},\sigma^{2}\right). \end{array}$$

Ref_*j*_ represents the mean score of the service where the ID is *j*. *r* represents the total number of service ratings. RoS_*j*_ is the score for *j*'s service. We need to set the value of *δ* to control the confidence interval. Filtering is more accurate if *δ* is a low value. However, this increases the complexity of processing the preferences. The specific PW is obtained using the following:

(11) $$\begin{array}{c} {\text{PW}}_{ij}=\{\begin{array}{cc} 0 & \left|\text{Ro}{\text{S}}_{j}-{\text{Ref}}_{j}\right|\leq f\left(\frac{\alpha }{2}\right)\\ C_{ij}-{\text{Ref}}_{j} & \text{else}. \end{array} \end{array}$$

PW_*ij*_ represents the *i*th consumer's PW for the *j*th service. We obtain a more objective service reputation rating after eliminating the personal preferences. The reputation rating of service *j* is calculated using the following:

(12) $$\begin{array}{c} \text{Ro}{\text{S}}_{j}=\frac{1}{r}\overset{r}{\underset{i=1}{\sum }}\left(\text{Ro}{\text{S}}_{ij}-{\text{PW}}_{ij}\right). \end{array}$$

If a consumer wants to access a service, the trust system provides a recommendation based on the reputation using (13). The reputation rating of each consumer is not the same because they have specific preferences, which also facilitates the punishment of malicious evaluations. RR_*ji*_ represents the reputation rating of service *j* recommended to consumer *i*:

(13) $$\begin{array}{c} {\text{RR}}_{ji}=\text{Ro}{\text{S}}_{j}+{\text{PW}}_{ij}. \end{array}$$

### 4.4. Dynamic Reputation Optimization Using a Market Mechanism

**Figure 4:**
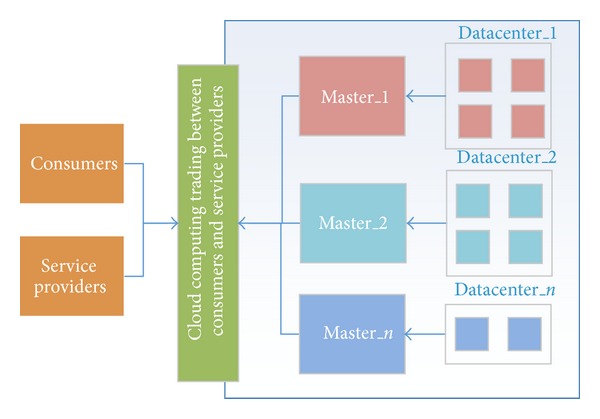
Cloud service market structure.

Cloud services are reliant on the dynamic and distributed cloud environment. Thus, a trust system needs to adapt to open and changing conditions. Service providers will change the configurations of their services to meet consumer demands, and the consumers will have variable service preferences. Different consumers use the same service, which runs in different data centers. We use a market mechanism [25] to construct a reputation optimization method that provides a better QoS. The cloud service market environment is shown in Figure 4. The data center, which is a distributed cloud resource provider, includes many nodes. The master node is the seller's agent, which is responsible for changing the service configuration and optimizing the service performance.

The market mechanism includes buyers and sellers. The buyers are consumers and the sellers are service providers in our method. We state the objectives of the buyers and sellers in the cloud market. The buyers' goal is to satisfy their personal demand for a service with a high reputation. The sellers' goal is to receive a better evaluation by optimizing their configuration to maximize the number of tasks completed successfully. The global objective of the market is to supply each buyer with a reliable service while minimizing the costs of the sellers.

The buyers and sellers have different demands, and the cloud service market is open and dynamic. We define the buyer's personal valuation using a utility function, which for buyer *b* using service *i* is defined as *P*_*b*_(*i*) = −*α*Cost(*i*) − *β*Time(*i*) + *γ*Profit(*i*), where Cost(*i*) and Time(*i*) represent the price and response time for service *i*, respectively, and Profit(*i*) is the payoff derived from the usage of service *i*. The seller's utility function is defined by *P*_*s*_(*i*) = *O*(*i*) − *C*(*i*), where *O*(*i*) represents the reputation and fees received for service *i* and *C*(*i*) is the cost of service *i*. The seller's objective is to maximize *P*_*s*_(*i*).

After each transaction, the sellers are rated based on their performance in the task allocated to them. The buyer decides this rating by comparing the actual service completion time with the expected time, as well as with other services. The global objective of the market is to maximize the number of services that are supplied satisfactorily by service providers. This objective is defined using the following:

(14) $$\begin{array}{c} G\left(i,j\right)=\mathrm{Max}\overset{n}{\underset{i=1}{\sum }}\overset{m}{\underset{j=1}{\sum }}\left(R_{ij}-A_{ij}\right), \end{array}$$

where *R*_*ij*_ is the reputation rating given by buyer *i* and *A*_*ij*_ is the actual performance of service *j*.  Algorithm 1 shows how the market transactions operate between buyers and sellers in pseudocode.

The buyer's timely and correct feedback facilitates the continuous optimization of the service in a dynamic market. If new services and buyers join the market, the system needs to be updated and new items will be available for the buyer to choose. After the service has been updated, a recommendation will be given to the buyer, but the reputation continues to be accumulated.

The market contains many submarkets. Submarkets are synchronized regularly using older market information, which reduces the frequency of management. A service can be present in different submarkets, which are distributed and flexible.

## 5. Experiments and Analysis

We produced a simulation program in Java to validate our reputation revision method. We simulated several service providers, which had different services with the same functions, as well as transaction behaviors of consumers. Our experiment comprised 500 consumers and four services, where each consumer rated the services they used after each transaction. The results were saved in the format shown in Table 3. To filter abnormal evaluations, our reputation revision method was used to cluster and mine the data in the transaction records after each round of transactions.

In our experiments, we tested two main hypotheses: (1) the consumer ratings of services follow a normal distribution; (2) the use of the approach described in Section 4 improves the accuracy of reputation ratings.

### 5.1. Distribution of Consumer Ratings

To verify the service score distribution, the consumers were divided into three types (i.e., bad, right, and good) who gave different ratings to services in our experiment. The bad consumers gave a score below the service's QoS level and the good consumers gave a score higher than the QoS level. The right consumers gave a score around the QoS level. All of the consumers gave random scores from the corresponding interval.

**Figure 5:**
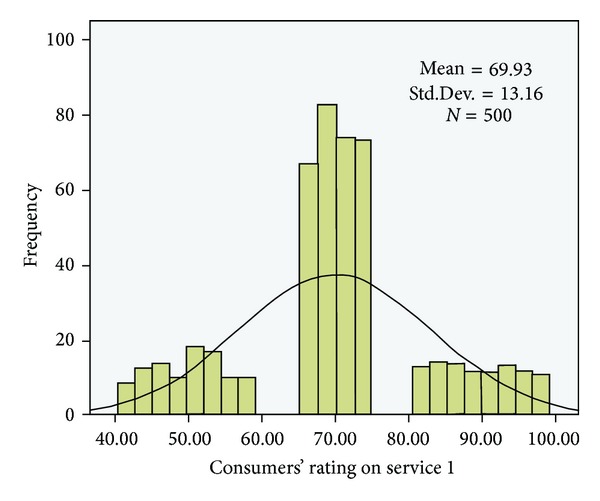
Distribution of rateValue for service 1 in the experiments (*N* = 500 users).

**Figure 6:**
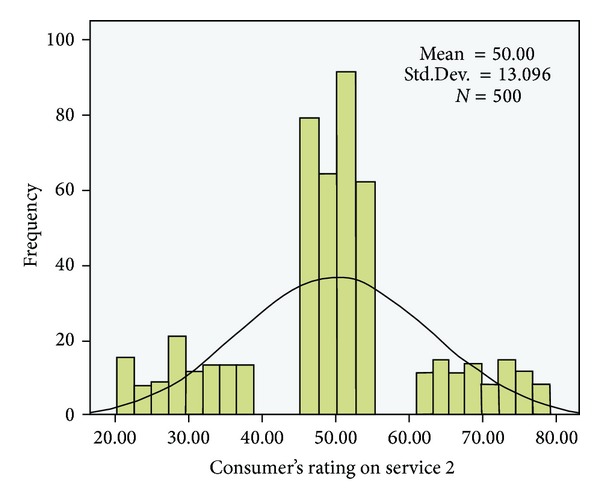
Distribution of rateValue for service 2 in the experiments (*N* = 500 users).

The following QoS metrics were considered in the experiments: cost, response time, and execution time. The cost value was selected randomly from the range [1, 10] $ and the response time was selected randomly from the range [1, 1000] s. The execution time was set as the functional unit/single unit. The experimental data were analyzed using SPSS. Figures 5 and 6 show the score distributions for the two services.

The results of the data analysis showed that the distribution of the service rating scores was not a strictly normal distribution. However, we used the confidence interval (the formula proposed in Section 4) to identify the right consumer ratings and to filter the bad and good ratings. Some value intervals were empty because the consumer ratings were based on the QoS level of the service and the consumers' preferences.

### 5.2. Reputation Revision

**Figure 7:**
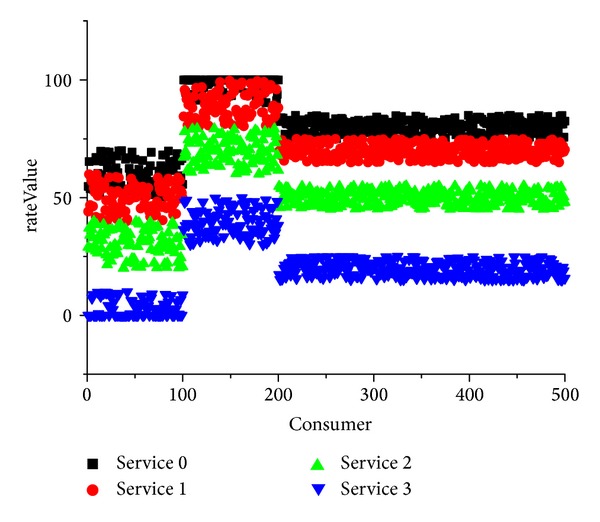
Ratings for four services given by 500 consumers.

**Figure 8:**
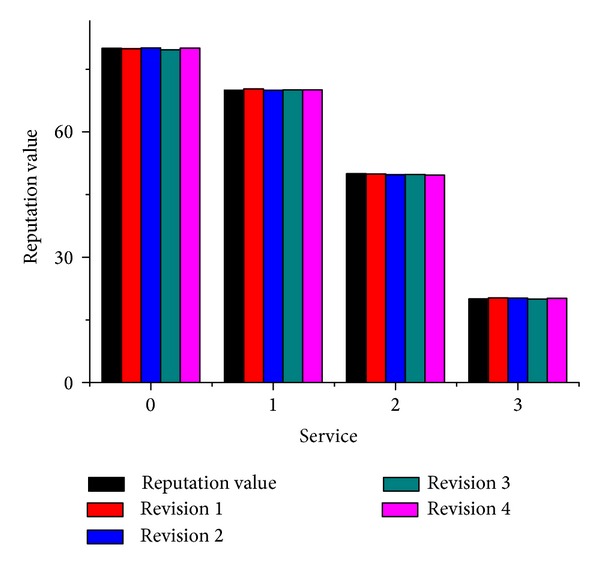
Four rounds of revised reputation.

In this experiment, we validated whether our method improved the accuracy of the service reputation ratings. In this experiment, four services provided the same function, but the QoS value belonged to different classes. The actual reputation of each service equaled the QoS value. We considered the revised reputation results for the four services after four cycles of revisions had been calculated.  Figure 7 shows the ratings made by 500 consumers for each service they used. Figure 8 shows the results after four cycles of reputation revision.

Figure 7 shows that the four services had different rateValue levels and the ratings also reflected the three different types of consumers, who had distinct scoring trends. Figure 8 demonstrates the accuracy and stability of the reputation revision method. The experiment showed that the revision method could identify abnormal reputation ratings, which were filtered from the overall evaluations to improve the accuracy of the service.

## 6. Conclusions

In this study, we developed a reputation revision method for cloud services based on the confidence interval of a normal distribution of ratings and a market mechanism. To address the problem of abnormal evaluations, we used prior knowledge to distinguish between different types of consumers before filtering dishonest ratings and setting the preferences for consumers.

There are still some limitations in the dynamic provision of services and reputation management [26] for each distributed submarket. The dynamic provision of services will add more complexity to the processing of historical data. It will also be necessary to consider reputation management in the distributed submarkets.

Future research should investigate the development of a mechanism for the dynamic evolution of reputation ratings and the application of this method to large-scale service-oriented systems. The growing number of services and users means that the configuration parameters of services and the user base are changing constantly, so reputation evaluations should be capable of evolving. We also expect that this method could be deployed in a real cloud service application system. Further verification of this reputation revision mechanism will help to identify new problems.

## Figures and Tables

**Algorithm 1:**
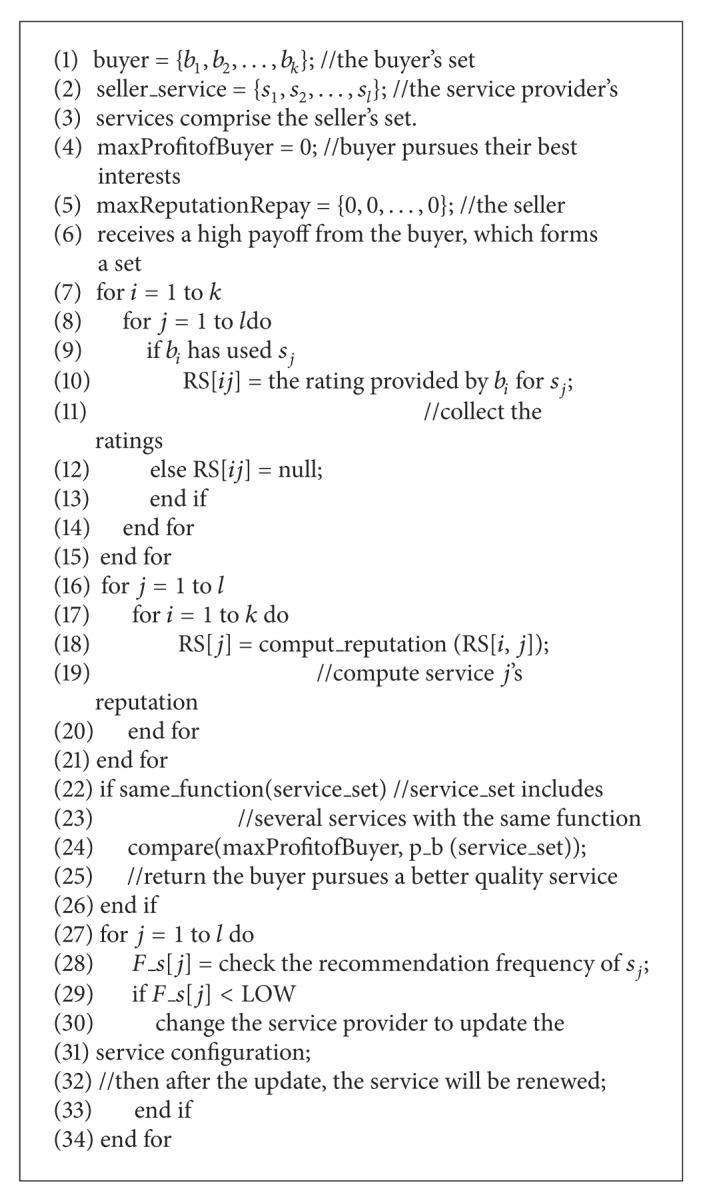
Trading between buyers and sellers.

**Table 1:** Quality of service parameters for a cloud service.

	*p*_1_	*p*_2_	*p*_3_	*p*_4_
*s*_1_	0.9	0.6	0.7	0.4
*s*_2_	0.8	0.7	0.6	0.3
*s*_3_	0.6	0.6	0.4	0.4
*s*_4_	0.5	0.2	0.2	0.8

**Table 2:** Reputations of services evaluated by consumers.

	*s*_1_	*s*_2_	*s*_3_	*s*_4_
*c*_1_	0.9	0.6	0.7	0.4
*c*_2_	0.8	0.7	0.6	0.3
*c*_3_	0.6	0.6	0.4	0.4
*c*_4_	0.5	0.2	0.2	0.8

**Table 3:** Quality of service (QoS) parameters for cloud services.

rateID	consumerID	serviceID	rateValue
int	int	int	float (calculated from the user's evaluation of the QoS attributes)
